# A Ceramic Diffusion Bonding Method for Passive LC High-Temperature Pressure Sensor

**DOI:** 10.3390/s18082676

**Published:** 2018-08-14

**Authors:** Chen Li, Boshan Sun, Yanan Xue, Jijun Xiong

**Affiliations:** 1 Key Laboratory of Instrumentation Science and Dynamic Measurement, Ministry of Education, North University of China, Taiyuan 030051, China; lichen@nuc.edu.cn (C.L.); sunbs333@163.com (B.S.); xueyananlu@163.com (Y.X.); 2 Science and Technology on Electronic Test & Measurement Laboratory, North University of China, Taiyuan 030051, China

**Keywords:** alumina ceramic, diffusion bonding, sensitive cavity, pressure sensor, high-temperature application

## Abstract

Alumina ceramic is a highly promising material for fabricating high-temperature pressure sensors. In this paper, a direct bonding method for fabricating a sensitive cavity with alumina ceramic is presented. Alumina ceramic substrates were bonded together to form a sensitive cavity for high-temperature pressure environments. The device can sense pressure parameters at high temperatures. To verify the sensitivity performance of the fabrication method in high-temperature environments, an inductor and capacitor were integrated on the ceramic substrate with the fabricated sensitive cavity to form a wireless passive LC pressure sensor with thick-film integrated technology. Finally, the fabricated sensor was tested using a system test platform. The experimental results show that the sensor can realize pressure measurements above 900 °C, confirming that the fabricated sensitive cavity has excellent sealing properties. Therefore, the direct bonding method can potentially be used for developing all-ceramic high-temperature pressure sensors for application in harsh environments.

## 1. Introduction

Recently, there has been an increased need for real-time, precise, and in situ pressure measurement in high-temperature environments, such as in combustion engines, geothermal sites, nuclear reactors, and space [1,2,3]. For example, for jet engines used in fighter aircraft, the temperature is usually within the range of 800–1450 °C [4], and in volcanic research, lava usually exhibits a non-Newtonian flow state between 800 and 1120 °C [5,6]. Traditional pressure sensors are based on silicon or silicon-on-insulator technology, and cannot operate above 450 °C [7,8,9]. Pressure sensors based on SiC for application in harsh environments have been extensively developed, and a 4H–SiC piezoresistive pressure sensor has been reported which can operate up to 800 °C [10,11,12,13,14]. However, their fabrication process was not as well developed as that of silicon, and the SiC substrate was very expensive. Furthermore, the pressure sensors are based on the piezoresistive effect, which cannot be used for wireless passive high-temperature sensor applications. Ceramic is a highly promising material for high-temperature sensors because of its excellent electrical, mechanical, and chemical properties, such as its strength, hardness, and erosion resistance. Recently, high-temperature pressure sensors based on ceramics have been demonstrated, but the fabrication method for these sensors is complex, and the sensor performance is poor. For example, LC passive pressure sensors are fabricated using low-temperature co-fired ceramic or high-temperature co-fired ceramic technology (which includes drying, cutting green tapes, silk screen printing, laminating, cutting, and high-temperature sintering). These pressure sensors have disadvantages regarding their fabrication process, especially the preparation of the sensitive cavity. The sensitive cavity can be fabricated only via filling with carbon film and laminating using green tapes, which complicates the fabrication process. The cavity can be easily broken or collapsed in the process of laminating and co-firing, which reduces the yield of the finished sensors. Importantly, the manufacturing process restricts the size of the sensitive cavity structure, which affects the sensitivity characteristics of the sensor. Additionally, the sensitive capacitance cavity can be fabricated using only certain green tapes (such as DuPont 951 or ferro) [15], and with the same rate of contraction as Ag conductive paste, which implies that the sensor cannot operate in high-temperature environments [16,17,18,19].

**Figure 1:**
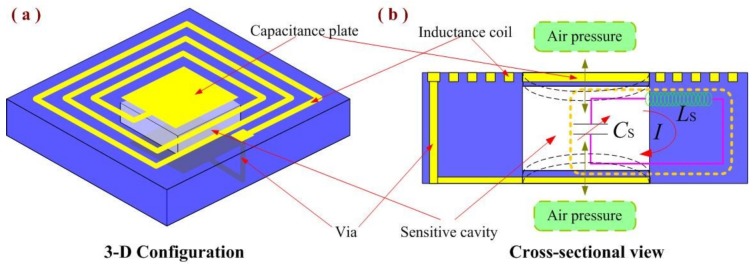
Structure of the wireless passive LC pressure sensor with the sensitive cavity. (**a**) Design schematic of the 3D configuration; (**b**) Cross-sectional view of the passive LC pressure sensor.

In this study, we developed a sensitive cavity fabrication method based on alumina ceramic for high-temperature pressure-sensor applications. It was fabricated via the direct bonding of alumina ceramic in a hot-pressing sintering environment and can be used for large cavity structures. Capacitor and inductor elements were integrated on the fabricated sensitive cavity based on alumina ceramic using thick-film integrated technology for the fabrication of an LC passive sensor. The structure of the wireless passive LC pressure sensor with a sensitive cavity is shown in Figure 1. Finally, a high-temperature, high-pressure test platform was set up, and the pressure sensitivity of the sensor was characterized as a function of the pressure in a high-temperature environment. Thus, the sensitivity performance of the fabricated sealing cavity was verified.

## 2. Experimental Procedure

Because the sensitive cavity is the core of the passive LC pressure sensor, its fabrication is highly important. To complete the fabrication of the sensitive cavity of the alumina-based capacitance element, we used a three-layer alumina ceramic substrate. The special experimental procedure is described in this section.

### 2.1. Pretreatment for Alumina Ceramic Plates

**Figure 2:**
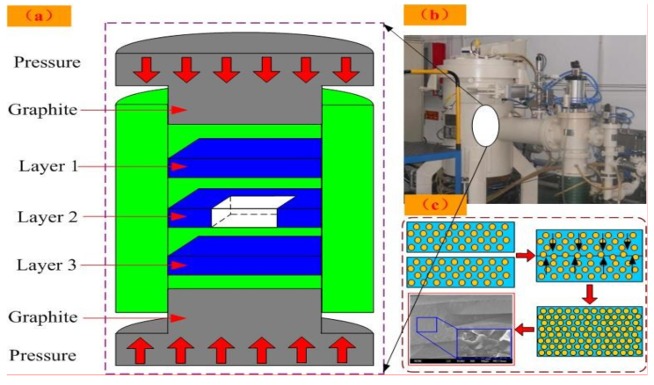
(**a**) Fabrication process for the sensitive cavity based on alumina ceramic; (**b**) Hot press furnace; (**c**) Special connecting process of the alumina ceramic.

It is important for the ceramic substrate to have a surface which is as flat and smooth as possible to obtain the best bond. First, three alumina ceramic layers were selected for the fabrication of the sensitive cavity. The ceramic plates (the Fifty-Four Research Institute of Electronics Technology Group Corporation, Shijiazhuang, China) were cut to 4 cm × 4 cm × 0.2 mm, and the properties are shown in Table 1. A square cavity was cut in the middle of the ceramic plate for the second layer, and the dimensions of the cavity were approximately 2 cm × 2 cm × 0.2 mm. Then, the surfaces of the substrates were ground and polished to remove any remnants of the substrate (such as oxidation film) and physical scars. After grinding and polishing, the grease on the surface of the alumina ceramic was removed using the ultrasonic vibration method. A cleaner surface enables a stronger bond for the ceramic substrate. Then, the cleaned alumina ceramic substrates were placed into an oven for drying. After the surface drying, the ceramic substrates were placed and laminated together in a graphite clamp in the designed order, in which layer 2 was the middle layer with the cavity, as shown in Figure 2a. For the graphite clamp, a flat surface forms the best bond. Then, the laminated substrates were placed into a high-temperature pressure-vacuum chamber for hot-pressing sintering, as shown in Figure 2b.

### 2.2. Hot-Pressing Sintering

When the preparation was completed, the hot press furnace (Shanghai Chen Xin Electric Furnce Co., Ltd., Shanghai, China) was heated under a vacuum condition to form the vacuum cavity. The atoms on the surfaces of the alumina ceramics were activated in the hot-pressing sintering environment to form bonds at the bonding interface and to realize mutual diffusion within the alumina ceramic. When the temperature reached 1500 °C, the samples were kept in the heat for 20 min, which caused them to be heated evenly and activated the atoms on the surface for bonding. Then, the bonded sample was cooled in the furnace to room temperature. To ensure good contact between the materials, we applied a pressure of approximately 5 MPa during the bonding process. After the hot-pressing sintering, the alumina ceramic substrates were bonded together closely, and the alumina ceramic-based sensitive cavity was formed. The special connecting process of the alumina ceramic is shown in Figure 2c.

### 2.3. Thinning

After hot-pressing sintering, the surface of the fabricated ceramic substrate with the sealed cavity was thinned to reduce the thickness of the sensitive membrane as much as possible. First, the fabricated ceramic substrate with sealed cavity was stuck onto the flat glass, using paraffin wax, and then the glass was fixed to the grinding disk of polishing machine by a vacuum clamp for thinning. Finally, the thickness of the sensitive membrane was about 0.1 mm.

### 2.4. Post-Fire Metallization

**Figure 3:**
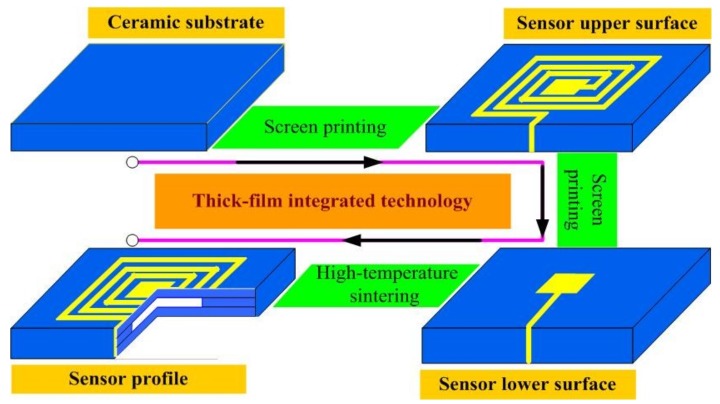
Post-fire metallization of the fabricated alumina ceramic sensitive cavity.

**Figure 4:**
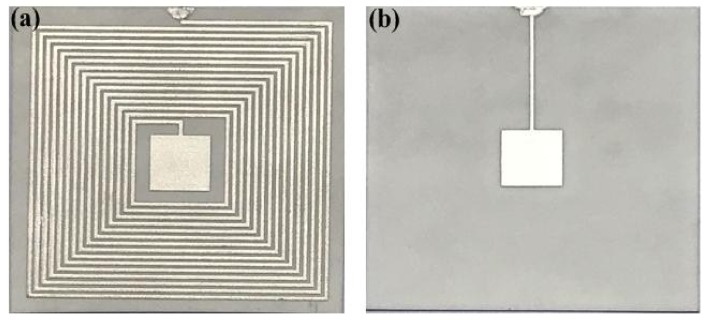
Sensor image. (**a**) Top view image; (**b**) bottom view image.

After the fabrication of the alumina ceramic-based sensitive cavity, to demonstrate the pressure sensitivity performance, electrical components were integrated on the sensitive cavity for the fabrication of the pressure sensor, as shown in Figure 1. The design of the passive LC pressure sensor comprised a fixed inductance coil and a flexible capacitor. These elements were connected to form a series LC resonant circuit, which was fabricated using thick-film integrated technology. To ensure the stability of the sensor in high-temperature environments, we used Pt paste for the fabrication of the electrical performance components. As shown in Figure 3, the fabrication process begins with the pretreatment of the fabricated ceramic substrate with a sealed cavity, in which the grease is removed using the ultrasonic vibration method; The next step is to fabricate the inductance elements and the capacitor top plate, using screen-printing technology, and then these are dried at 150 °C for 20 min; Further, the capacitor bottom plate is integrated on the sensor lower surface; And finally, the screen-printed alumina ceramic substrate is sintered in a high-temperature furnace for a total firing time of approximately 3 h at a peak temperature of 1500 °C to allow the Pt ink to cure. The top-view image and bottom-view image of the fabricated sensor are shown in Figure 4.

## 3. Measurement and Results

**Figure 5:**
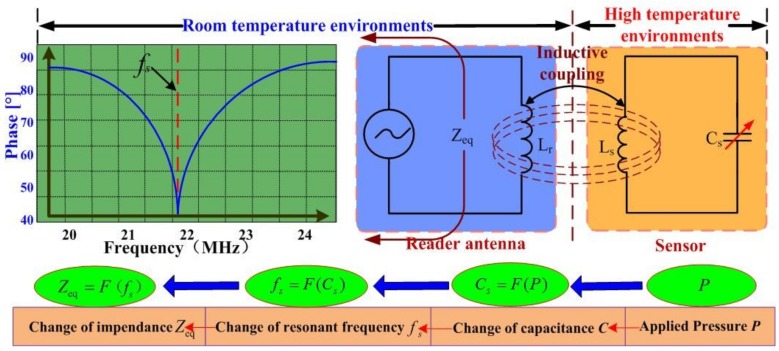
High temperature pressure-sensitive principle of sensor.

Figure 5 shows the high temperature pressure-sensitive principle of the sensor. When air pressure is applied to the sensitive cavity at a high temperature, the resonant frequency of the sensor changes; this is wirelessly detected by the external reader antenna. Then, the in situ pressure signal is transmitted from the high-temperature environment to a room-temperature environment through the inductance element of the sensor and antenna.

As shown in Figure 5, the equivalent impedance (*Z_eq_*) viewed from the reader antenna can be expressed as follows [20]:

(1) $$Z_{eq}=j2\pi fL_{r}[1+\frac{k^{2}(f/f_{s})}{1-{\left(f/f_{s}\right)}^{2}+jf/(f_{s}Q)}]=F(f_{s})$$

where *L_r_* is the inductance of the reader antenna; *k* is the coupling efficient between the reader antenna and sensor; *Q* is the quality factor of the sensor; *f* is the excitation frequency; and *f_s_* is the resonant frequency of the sensor, which can be expressed as follows:

(2) $$f_{S}=1/(2\pi \sqrt{L_{S}C_{S}})=F(C_{s})$$

where *L_s_* and *C_s_* are the inductance and capacitance of the sensor, respectively. Then, the capacitance *C*_s_ can be expressed as follows [15]:

(3) $$C_{s}\approx \frac{\varepsilon_{0}a^{2}}{t_{g}+\left(2t_{m}/\varepsilon_{r}\right)}\cdot \frac{\tanh^{-1}\left(\sqrt{\left(3Pa^{4}(1-v^{2})\right)/\left(16E{\left(t_{m}\right)}^{3}(t_{g}+(2t_{m}/\varepsilon_{r}))\right)}\right)}{\sqrt{\left(3Pa^{4}(1-v^{2})\right)/\left(16E{\left(t_{m}\right)}^{3}(t_{g}+(2t_{m}/\varepsilon_{r}))\right)}}=F(P)$$

where $\varepsilon_{0}$ and $\varepsilon_{r}$ are the vacuum dielectric constant and the relative permittivity of the alumina ceramic; *a* denotes the length of the capacitance plate, *ν* stands for Poisson’s ratio; *t_g_* and *t_m_* represent the cavity thickness and sensitive diaphragm thickness, respectively; and *P* is the applied pressure on the sensitive diaphragm.

**Figure 6:**
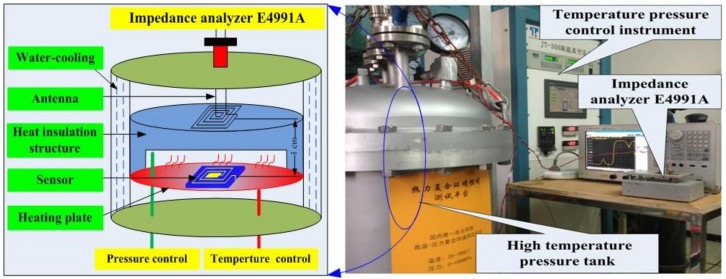
Side view of the measurement system platform.

To investigate the pressure sensitivity characterization of the cavity in high-temperature environments, the sensitivity of the fabricated sensor as a function of the pressure at a high temperature was measured using a high-temperature pressure measurement system comprising a high-temperature pressure tank, an E4991A impedance analyzer, and a temperature–pressure control instrument, as shown in Figure 6. The sensor and antenna are separated by 1 cm through the heat insulation structure, which was placed in the high-temperature pressure tank. The E4991A impedance analyzer can acquire the impedance phase and resonant frequency wireless readout from the test antenna. The temperature–pressure control instrument can accurately control the temperature and pressure of the high-temperature pressure tank, and the high-temperature pressure tank can provide a temperature–pressure environment with a maximum temperature of 900 °C and a maximum pressure of 3 bar.

**Figure 7:**
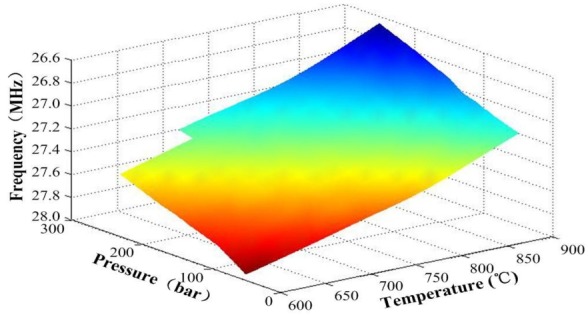
Resonant frequency of the sensor as a function of pressure and temperature.

To investigate the sensitivity characterization of the fabricated cavity under high temperatures, the pressure in the tank was varied between 0.20 and 2.80 bar above 600 °C. The changes in the sensor’s resonant frequencies when different temperatures and pressures were applied on the sensor are shown in Figure 7. In addition, in Figure 7, we can see the resonant frequency of the sensor gradually decreased as the pressure and temperature increased.

**Figure 8:**
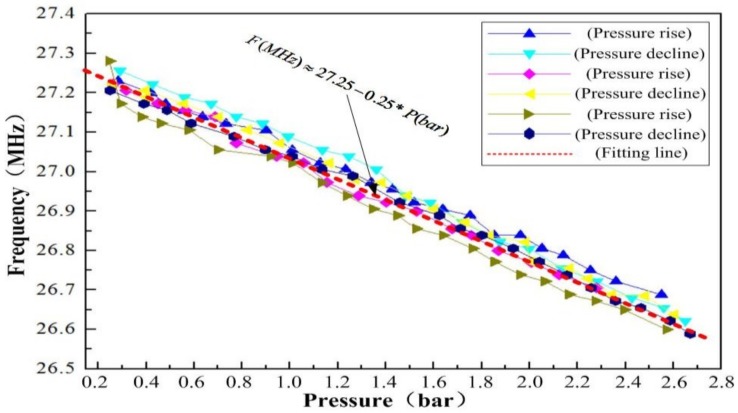
Resonant frequency of the sensor as a function of the pressure at 900 °C.

Figure 8 shows the several measured results of the sensor with respect to the pressure at 900 °C. The resonant frequencies of the sensor were approximately 27.25 and 26.60 MHz at 0 and 2.60 bar, respectively. As shown in Figure 8, the resonant frequency of the sensor decreased linearly as the applied pressure increased at 900 °C, and the pressure sensitivity of the sensor was only approximately −0.25 MHz/bar. The hysteresis error and repeatability error of the sensor were 9.3% and 13% approximately, as a function of pressure at 900 °C. These measurement results indicate that the cavity of the sensor is sealed, which can increase the sensitivity to pressure in high-temperature environments, and that the sensitive cavity fabrication method for the pressure sensor can be applied for in situ pressure measurement in high-temperature environments.

## 4. Conclusions

We describe a fabrication method for a pressure-sensitive cavity based on alumina ceramic, which can be used for the fabrication of high-temperature pressure sensors. The method has the characteristics of simple operation, a high molding rate, and ease of use. The sensitive cavity was fabricated via direct bonding of alumina ceramic in a hot-pressing sintering environment, and a wireless passive LC high-temperature pressure sensor based on the fabricated sensitive cavity was fabricated using thick-film integrated technology. The experimental results show that the sensor can perform in situ pressure measurements in high-temperature environments and has good pressure sensitivity of up to −0.25 MHz/bar at 900 °C. Therefore, it is a promising pressure measurement sensor for high-temperature environments. This research can provide a useful reference for the fabrication of alumina ceramic high-temperature pressure sensors and provides a new manufacturing process for high-temperature alumina ceramic sensors with sealed cavities.

## Figures and Tables

**Table 1:** Properties of the ceramic plate.

Property	Value
Composition	Al_2_O_3_
Density	3.89 g/cm^3^
Surface roughness	<30 nm
